# A Panel of Circulating MicroRNAs Detects Uveal Melanoma With High Precision

**DOI:** 10.1167/tvst.8.6.12

**Published:** 2019-11-14

**Authors:** Mitchell S. Stark, Elin S. Gray, Timothy Isaacs, Fred K. Chen, Michael Millward, Ashleigh McEvoy, Pauline Zaenker, Melanie Ziman, H. Peter Soyer, William J. Glasson, Sunil K. Warrier, Andrew L. Stark, Olivia J. Rolfe, Jane M. Palmer, Nicholas K. Hayward

**Affiliations:** 1 The University of Queensland Diamantina Institute, The University of Queensland, Dermatology Research Centre, Brisbane, Queensland, Australia; 2 School of Medical and Health Sciences, Edith Cowan University, Joondalup, Western Australia, Australia; 3 Centre for Ophthalmology and Visual Science, The University of Western Australia, Crawley, Western Australia, Australia; 4 Department of Ophthalmology, Royal Perth Hospital, Perth, Western Australia, Australia; 5 Perth Retina, West Leederville, Western Australia, Australia; 6 Lions Eye Institute, Nedlands, Western Australia, Australia; 7 School of Medicine and Pharmacology, The University of Western Australia, Crawley, Western Australia, Australia; 8 Department of Medical Oncology, Sir Charles Gairdner Hospital, Nedlands, Western Australia, Australia; 9 School of Biomedical Science, The University of Western Australia, Crawley, Western Australia, Australia; 10 Department of Dermatology, Princess Alexandra Hospital, Brisbane, Queensland, Australia; 11 Queensland Ocular Oncology Service, The Terrace Eye Centre, Brisbane, Queensland, Australia; 12 QIMR Berghofer Medical Research Institute, Brisbane, Queensland, Australia

**Keywords:** uveal, melanoma, miRNA, microRNA, biomarker, diagnostic, prognostic

## Abstract

**Purpose:**

To determine if a circulating microRNA (miRNA) panel could be used to distinguish between uveal melanoma and uveal nevi.

**Methods:**

We report on a multicenter, cross-sectional study conducted between June 2012 and September 2015. The follow-up time was approximately 3 to 5 years. Blood was drawn from participants presenting with a uveal nevus (*n* = 10), localized uveal melanoma (*n* = 50), or metastatic uveal melanoma (*n* = 5). Levels of 17 miRNAs were measured in blood samples of study participants using a sensitive real-time PCR system.

**Results:**

A panel of six miRNAs (miR-16, miR-145, miR-146a, miR-204, miR-211, and miR-363-3p) showed significant differences between participants with uveal nevi compared with patients with localized and metastatic uveal melanoma. Importantly, miR-211 was able to accurately distinguish metastatic disease from localized uveal melanoma (*P* < 0.0001; area under the curve = 0.96). When the six-miRNA panel was evaluated as a group it had the ability to identify uveal melanoma when four or more miRNAs (93% sensitivity and 100% specificity) reached or exceeded their cut-point.

**Conclusions:**

This miRNA panel, in tandem with clinical findings, may be suited to confirm benign lesions. In addition, due to the panel's high precision in identifying malignancy, it has the potential to augment melanoma detection in subsequent clinical follow-up of lesions with atypical clinical features.

**Translational Relevance:**

Uveal nevi mimic the appearance of uveal melanoma and their transformation potential cannot be definitively determined without a biopsy. This panel is most relevant at the nevus stage and in lesions with uncertain malignant potential as a companion diagnostic tool to assist in clinical decision-making.

## Introduction

Melanoma of the uveal tract (iris, ciliary body, and choroid) is the most common intraocular malignancy in adults. Uveal melanoma (UM) can form in the iris, located at the front of the eye, though most are located in the posterior segment of the eye (ciliary body or choroid), with the latter considered more malignant. Due to their location, posterior UM are often detected later and as such may be more prone to metastasis, especially when carrying specific chromosomal aberrations (loss of chromosome 3 and gain of chromosome 8q) determined cytogenetically following intraocular biopsy.^1^ Several benign tumors (e.g., hemangiomas and uveal nevi) can mimic UM. Importantly, all melanocytic nevi have the potential to transform into melanoma, with the transformation rate of choroidal nevi being higher than that of cutaneous nevi (∼1/200,000).^2^,^3^ It has been estimated that progression of a choroidal nevus to UM occurs in approximately 1 in 9000 cases.^4^ However, if high-risk clinical features are identified, the risk for transformation is 69%.^3^ Therefore, close monitoring by an ocular oncologist is required to detect the early signs of malignancy.

Circulating biomarkers (proteins, cell-free DNA, and microRNAs [miRNA]) have been intensely studied in a wide range of malignancies as a minimally invasive method of cancer detection and for predicting prognosis. In UM, serum levels of DJ-1 (PARK7) were found to be significantly associated with choroidal nevus growth.^5^ Circulating *GNAQ*/*GNA11* mutations (found in ∼83% of UM^6^) have been detected in plasma of metastatic UM patients^7^,^8^; however, they are rarely detectable in patients with localized disease.^9^ In recent years, circulating miRNA has been found to be a highly sensitive and specific method of identifying underlying malignancy in cutaneous and UM.^10^–^14^

MiRNAs are short (∼20–22 nt), noncoding RNAs that are readily detectable in tissues and blood. We previously identified a panel of melanoma-related miRNAs that offered superior sensitivity to currently used serologic markers for cutaneous melanoma progression, recurrence, and survival.^10^ We therefore sought to assess this panel in serum from patients with choroidal nevi, localized UM, and metastatic UM.

We herein present a panel of circulating miRNA that can accurately distinguish choroidal nevi from localized UM, as well as detect metastatic disease with high sensitivity and specificity.

## Materials and Methods

### Patient Specimen Details

A prospectively collected sample of patients was ascertained at the Queensland Ocular Oncology Service, Brisbane, Queensland, Australia and the Lions Eye Institute and Royal Perth Hospital, Perth, Western Australia, between June 2012 and September 2015. All patients gave written informed consent (in accordance with the Declaration of Helsinki) under approved protocols governed respectively by the Human Research Ethics Committee of the QIMR Berghofer Medical Research Institute (No. P1237), Edith Cowan University (No. 11543), and Sir Charles Gardner Hospital (No. 2013-246). All serum samples were collected in 8.5-mL BD serum separator tubes (SST; BD, North Ryde, Australia). Blood was left to clot for 30 minutes at room temperature, then centrifuged for 10 minutes at 1500 *g*. The serum supernatant was then aliquoted into 1.5-mL cryovials and stored at −80°C until further use. Patient demographics are given in Table 1.

**Table 1:** Descriptive Statistics of All Serum Cohorts Used Within the Study

Prognostic Factors	Terrace Eye Centre, *n* (%)	Lions Eye Institute and Royal Perth Hospital, *n* (%)	Combined Cohorts, *n* (%)
Totals	43 (100)	22 (100)	65 (100)
Sex			
Male	17 (40)	14 (64)	31 (48)
Female	26 (60)	8 (36)	34 (52)
Age at blood draw			
20–30	2 (5)	0 (0)	2 (3)
31–40	1 (2)	0 (0)	1 (2)
41–50	5 (12)	3 (14)	8 (12)
51–60	8 (19)	11 (50)	19 (29)
61+	27 (63)	8 (36)	35 (54)
Status at blood draw			
Uveal naevus	10 (23)	0 (0)	10 (15)
Localized	31 (72)	19 (86)	50 (77)
Metastatic	2 (5)	3 (14)	5 (8)
Status at last follow-up			
Alive NSR	28 (65)	13 (59)	41 (63)
Alive status unknown	6 (14)	0 (0)	6 (9)
Alive with melanoma	3 (7)	7 (32)	10 (15)
Death from melanoma	5 (12)	2 (9)	7 (11)
Unknown	1 (2)	0 (0)	1 (2)

NSR, no sign of recurrence.

### Clinical Diagnostic Criteria

Each lesion was assessed using a combination of fundoscopy, Optos widefield retinal imaging (Optos Inc., Marlborough, MA), optical coherence tomography, and B-scan ultrasonography. The diagnosis of choroidal melanoma was based on the presence or absence of features conferring high risk of growth in a choroidal lesion.^3^ These features include lesion height of more than 2 mm, visual symptoms (e.g., visual loss, photopsia), low echogenicity on B-scan, the presence of subretinal fluid and/or orange lipofuscin, close proximity to the optic nerve (≤3 mm), and absence of surrounding halo depigmentation or absence of overlying retinal pigment epithelium (RPE) alterations (e.g., drusen, atrophy, hyperplasia, detachment, fibrous metaplasia).^3^ Choroidal lesions with three or more of these features were diagnosed as choroidal melanoma. Asymptomatic choroidal lesions with minimal elevation (height <2 mm), high echogenicity, and overlying RPE changes but no subretinal or orange pigment were diagnosed as choroidal nevi.

### Total RNA Extraction

Extraction of total RNA from serum was performed following the manufacturer's protocol using miRNeasy Serum/Plasma Kits (QIAGEN, Hilden, Germany) as previously described.^15^ A synthetic miRNA mimic (miRNeasy Serum/Plasma Spike-In Control: C. elegans miR-39 miRNA mimic, Cat No 219610; QIAGEN) was spiked into each sample to allow for normalization of expression data.

### MicroRNA Panel Selection

The MELmiR-17 panel described previously^10^,^16^ was selected for analysis because all members of this panel were found to be expressed in UM cell lines (see Results).

### Reverse Transcription, Preamplification, Taqman Assays, and Fluidigm Real-Time PCR

A custom Taqman assay combined with a sensitive method of detection (Fluidigm; HD Biomark, South San Francisco, CA) was used as described.^10^,^15^ Briefly, a custom reverse transcription (RT) primer pool consisting of equal amounts of miRNA-specific RT primers contained within each TaqMan Assay (Life Technologies, Carlsbad, CA); miR-16 (000391), miR-145-5p (002278), miR-146a-5p (000468), miR-204-5p (000508), miR-211-5p (000514), miR-363-3p (001271), miR-506-3p (001050), miR-508-3p (001052), miR-508-5p (002092), miR-509-3p (002236), miR-509-5p (002235), miR-513b (002757), miR-513c-5p (002756), miR-514a-3p (001147), miR-4487 (462492_mat), miR-4706 (464518_mat), and miR-4731-5p (464084_mat) along with cel-miR-39 (000200; serum spiked-in control) plus an additional pool of the corresponding TaqMan MicroRNA Assay (Pre-Amp Primer Pool) were used to preamplify the RT reaction.

### qRT-PCR Analysis

Expression of the MELmiR-17 panel was assayed in each sample with four technical replicate Taqman assays. Real-time expression data were extracted and analyzed as previously described.^15^

### Statistical Methods

GraphPad Prism version 8.0.0 for Windows (San Diego, CA) was used for all statistical analysis. Each miRNA was assessed for expression variance using a one-way ANOVA or Kruskal-Wallis test. Next, the miRNA that were deemed significant (*P* ≤ 0.05) were corrected for multiple comparisons using a two-stage, linear, step-up procedure of Benjamini, Krieger, and Yekutieli, which controls the false discovery rate (FDR) and provides a corrected *P* value. Kaplan-Meier survival curve analysis of each miRNA was also assessed across sample groups. Mann-Whitney *U* test was performed for pair-wise comparisons of recurrence versus no recurrence. Predictive ability of each miRNA was evaluated using receiver operating characteristic (ROC curve) and area under the curve (AUC) or AUROC (GraphPad Prism 8).

### Diagnostic Score Assignment

AUC scores of 0.70 or more were deemed to be diagnostically useful.^17^ The data presented in the Figure indicated increased expression (i.e., lower median-normalized Ct value) was associated with disease progression. The miRNAs that had an AUC of 0.70 or more when nevi were compared with localized UM were interrogated further to classify the median-normalized Ct values as ‘high' or ‘low' expression (interpretation of the median normalized Ct expression values used to determine ROC curves were evaluated using an arbitrary cut point of ≥85% sensitivity). If the expression value in each sample reached or exceeded this value then it was counted as positive for UM (independent of disease status). This method of determining positivity was performed for each of the six miRNAs. For each sample, the sum of all positive values ranged from 1 to 6. This sum is the diagnostic score for that individual sample.

**Figure:**
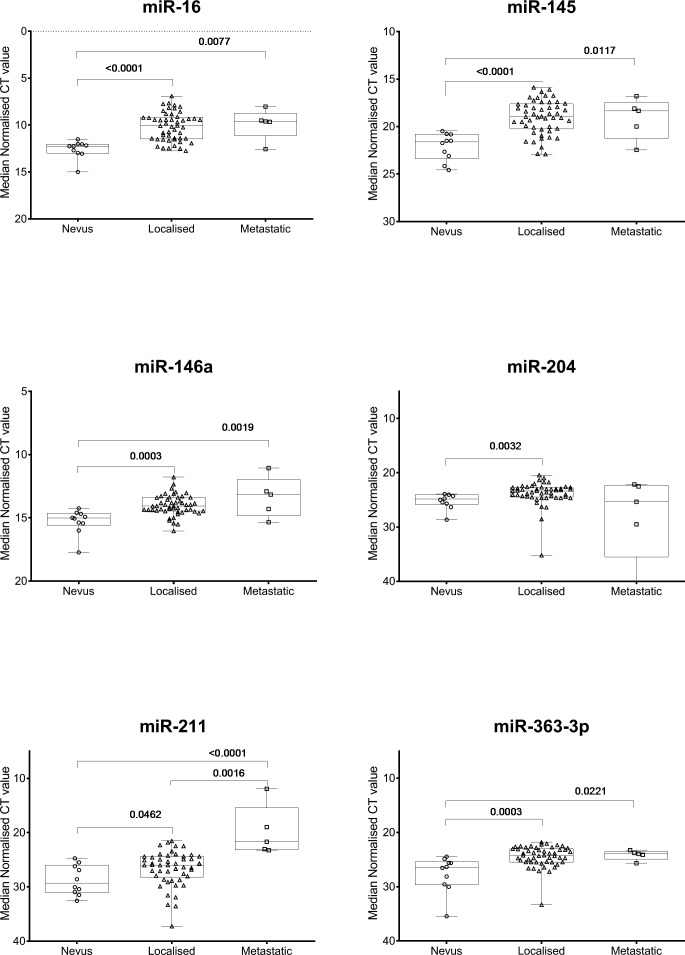
Box and Whisker plots (minimum to maximum) collate all data points represented in Table 2 for the six-miRNA panel that were significantly different (ANOVA P < 0.05) across the cohorts of uveal nevi, localized UM, and metastatic UM. The associated corrected P values from Table 2 are illustrated here. These data indicate the circulating levels of members of the miRNA panel increase significantly with disease progression.

### Diagnostic Score Evaluation

The diagnostic scores were evaluated using a 2 × 2 confusion matrix method and the following formula: positive predictive value (PPV) or precision = true positive (TP)/(TP + false positive [FP]); negative predictive value (NPV) = true negative (TN)/(false negative [FN] + TN); sensitivity = TP/(TP + FN); specificity = TN/(FP + TN) false-positive rate = 1 – specificity; false-negative rate = 1 – sensitivity; likelihood ratio positive = sensitivity/1-specificity; likelihood ratio negative = 1-sensitivity/specificity; diagnostic odds ratio (DOR) = (TP/FN)/(FP/TN).

## Results

### A ‘Melanoma-Related' miRNA Panel is Expressed in Both Cutaneous and Uveal Melanoma

Prior to evaluating the MELmiR-17 panel in patient serum, we first confirmed that the miRNAs were expressed in UM cell lines (*n* = 6). Importantly, all members of this panel were found to be expressed in at least one of the cell lines (92.1, MEL202, MEL270, MEL285, MEL290, OMM1) previously described and assessed via a miRNA microarray^16^ (see Supplementary Table S1^16^). In our prior study, miR-211 was the top-ranked miRNA (277-fold average higher expression in 71% or *n* = 39/55) when cutaneous melanomas were compared with other solid cancer types (e.g., breast, prostate, colorectal, etc.).^16^ Consistent with the reported high expression in cutaneous melanoma, miR-211 was present in 5/6 (83%) UM cell lines, and was highly expressed.^16^

### A Panel of Six miRNAs Identifies Localized and Metastatic Uveal Melanoma With High Sensitivity and Specificity in Patient Sera

Expression of the MELmiR-17 panel was measured in serum samples from two independent, prospectively collected patient cohorts (Table 1). Study participants had a single blood drawn at presentation with clinical signs of choroidal nevi (*n* = 10), localized UM (*n* = 50), or metastatic UM (*n* = 5). In miRNA derived from the patient serum, 11 of 17 miRNAs had detectable expression in all specimen types (Fig. and Supplementary Fig. S1). No expression was detected for miR-506-3p, miR-508-3p, miR-508-5p, miR-513b, miR-513c, or miR-514a. Of 11 detected miRNAs, six (miR-16, miR-145, miR-146a, miR-204, miR-211, and miR-363-3p) showed significant differences (ANOVA; *P* < 0.05) across the cohort. Variation in levels of the remaining five expressed miRNAs (miR-509-3p, miR-509-5p, miR-4487, miR-4706, and miR-4731) did not reach significance (ANOVA; *P* > 0.05) across the sample types in this study and were thus not explored further. Interestingly, all five of these “nonsignificant” miRNAs were highly significant in our prior study investigating cutaneous melanoma,^10^ which suggests that these are more relevant in cutaneous than UM.

The miRNAs that reached or exceeded the significance threshold (*P* < 0.05) were next assessed using a multiple comparison analysis. The Figure and Table 2 provide a summary of the multiple comparisons (uveal nevi versus patients with localized UM, nevi versus metastatic UM, and localized versus metastatic UM) and associated corrected *P* values (*P*[cor]). All six of the significant miRNAs by ANOVA remained significant (*P*[cor] = 0.0462 − *P*[cor] < 0.0001) when uveal nevi were compared with localized UM, with miR-16 and miR-145 being highly significantly different (*P*[cor] < 0.0001). Next, all ANOVA significant miRNAs (except miR-204) again showed significance (*P*[cor] = 0.0221 − *P*[cor] < 0.0001) when uveal nevi were compared with metastatic UM, with miR-211 being highly significant (*P*[cor] < 0.0001). Notably, when localized UM was compared with metastatic UM, miR-211 was the only member of the panel to reach significance (*P*[cor] = 0.0016) (Fig. and Table 2).

**Table 2:** Table Provides a Summary of the One-Way ANOVA (Kruskal-Wallis test) Performed to Determine Which miRNA Showed Significant (P < 0.05) Variance Across the Cohorts

Comparison	Test	miR-16	miR-145	miR-146a
All cohorts (naevi, localized, metastatic	Kruskal-Wallis test	<0.0001	<0.0001	0.0001
Naevi (*n* = 10) vs. localized (*n* = 50)	Benjamini, Krieger, and Yekutieli method (False Discovery Rate corrected *P *value)	<0.0001	<0.0001	0.0003
	AUROC score	0.92 (0.85, 0.99)	0.91 (0.84, 0.99)	0.88 (0.79, 0.98)
Naevi (*n* = 10) vs. metastatic (*n* = 5)	Benjamini, Krieger, and Yekutieli method (False Discovery Rate corrected *P* value)	0.0077	0.0117	0.0019
	AUROC score	0.88 (0.65, 1.0)	0.88 (0.65, 1.0)	0.86 (0.63, 1.0)
Localized (*n* = 50) vs. metastatic (*n* = 5)	Benjamini, Krieger, and Yekutieli method (False Discovery Rate corrected *P* value)	0.9353	0.9514	0.3587
	AUROC score	nd	nd	nd

ns, nonsignificant or *P* > 0.05; nd, not defined.

Multiple testing was performed to correct for false discovery rate (Benjamini, Krieger, and Yekutieli Method, corrected *P* values). AUROC analyses were performed in each cohort comparison for the 6 significant miRNAs. AUC scores with confidence intervals are shown. Brackets represent the 95%CI.

**Table 2:** Extended

Comparison	Test	miR-204	miR-211	miR-363-3p
All cohorts (naevi, localized, metastatic	Kruskal-Wallis test	0.0055	0.0003	0.0003
Naevi (*n* = 10) vs. localized (*n* = 50)	Benjamini, Krieger, and Yekutieli method (False Discovery Rate corrected *P v*alue)	0.0032	0.0462	0.0003
	AUROC score	0.81 (0.70, 0.93)	0.71 (0.55, 0.88)	0.86 (0.75, 0.96)
Naevi (*n* = 10) vs. metastatic (*n* = 5)	Benjamini, Krieger, and Yekutieli method (False Discovery Rate corrected *P* value)	0.4009	<0.0001	0.0221
	AUROC score	0.52 (0.12, 0.92)	1.0 (1.0, 1.0)	0.92 (0.76, 1.0)
Localized (*n* = 50) vs. metastatic (*n* = 5)	Benjamini, Krieger, and Yekutieli method (False Discovery Rate corrected *P* value)	0.2329	0.0016	0.9820
	AUROC score	nd	0.96 (0.90, 1.0)	nd

In comparisons presented in the Figure, miRNA expression levels significantly increase with tumor progression (uveal nevi versus localized UM), which indicate these may be diagnostically useful in initial diagnosis and to monitor disease progression using serial blood draws. To determine the discriminatory power of each of the six miRNAs, AUROC analysis was next performed for uveal nevi versus localized UM (Table 2 and Supplementary Fig. S2) and nevi versus metastatic UM (Table 2 and Supplementary Fig. S3). All miRNAs could be considered diagnostically useful for identifying malignancy, with AUC scores ranging from 0.7 to 1.0. Importantly, miR-211 was able to accurately distinguish metastatic disease from localized UM (*P* < 0.0001; AUC = 0.96) (Supplementary Fig. S4).

The sensitivity and specificity of the miRNA panel was then assessed by assigning a diagnostic score to the data. The expression values graphed in the Figure were used to observe the direction of the data (i.e., higher expression in localized UM versus uveal nevi). The cut-points in the AUROC datasets (not shown) were identified which allowed the expression values to be categorized as positive or negative for UM (see Materials and Methods). Upon applying the derived diagnostic score, the miRNA panel can then be evaluated as a group. Based upon these discovery data, the miRNA panel had the ability to identify UM (localized and metastatic), when four or more miRNAs (93% sensitivity and 100% specificity) reached or exceeded their cut-point (Table 3). The FP and FN rate was used to determine the lowest diagnostic score possible for the miRNA panel while still maintaining very high sensitivity and specificity. For example, when a specimen had a score of 3 or more, the sensitivity was high (93%) but specificity was unacceptably low (60%). Likewise, if ≥5 were included, this achieved 100% specificity but reduced the sensitivity to 82%. Following on from this interpretation, a diagnostic score can then be applied to each sample, which ranges from 0 to 3 (lower likelihood of UM) and 4 to 6 (higher likelihood of UM).

**Table 3:** Results of the Diagnostic Test Evaluations Generated When Uveal Nevi is Compared With Localized and/or Metastatic Melanoma

miRNA Panel	Uveal Naevi vs. Localized or Metastatic Melanoma	Uveal Naevi vs. Localised or Metastatic Melanoma	Uveal Naevi vs. Localised or Metastatic Melanoma
Diagnostic score	≥3	**≥4**	≥5
Sensitivity, %	93	**93**	82
Specificity, %	60	**100**	100
False-positive rate, %	40	**0**	0
False-negative rate, %	7	**7**	18
PPV, %	93	**100**	100
NPV, %	60	**76**	50
Likelihood ratio positive	2	**∼20**	∼18
Likelihood ratio negative	0.13	**0.07**	0.19
DOR	19	**∼240**	∼93

The associated results are presented for the diagnostic scores of ≥3, ≥4, and ≥5 which is the total number of miRNAs (of 6) expressed per sample that reach or exceed the cut point of ≥85% sensitivity (see Materials and Methods). The bolded results represent the diagnostic score (≥4) that gave the highest sensitivity (93%) and specificity (100%). The likelihood ratios (positive) and diagnostic odds ratios shown as approximate were calculated by adding 0.5 to the 2 × 2 confusion matrix as the number of false-positives was zero.

### Recurrence and Overall Survival

In the Queensland Ocular Oncology Service cohort (Table 1) with 4- to 5-year follow-up data available, one of 10 pigmented choroidal lesion demonstrated growth. In study participants presenting with localized UM, five of 31 (16%) developed liver metastases, with two of five confirmed deaths from UM. We next assessed the Lions Eye Institute and Royal Perth Hospital cohort with up to 4-year follow-up information available. In participants presenting with localized UM, three of 19 (16%) had a localized recurrence, with one participant progressing further to metastatic disease. Overall, five of 19 (26%) participants from this cohort developed liver (4/5, 80%) or lung (1/5, 20%) metastases with two of five confirmed deaths from UM.

The miRNA panel was next analyzed for association with recurrence (local or metastatic) and overall survival (OS). With a single blood draw, no statistically significant association with recurrence or time to recurrence (Mann-Whitney *U* test and Kaplan Meier survival curve analysis respectively; data not shown) was evident in either cohort. Next the six-miRNA panel was assessed for OS using optimal cut points in the dataset to determine “high” and “low” expression. Interestingly, low-circulating miR-204 expression was found to be significantly (Log rank, *P* = 0.014) associated with poor overall survival as compared with high-circulating expression levels (Supplementary Fig. S5). All other miRNA showed nonsignificant associations with OS, however high miR-211 expression did have a nonsignificant trend toward poor OS (Supplementary Fig. S5).

A paired blood draw was available for one individual who presented with localized UM. With blood draws approximately 1 year apart, expression levels were increased at follow-up for miR-211 but not for any of the other miRNAs (data not shown). Follow-up information from this individual revealed a localized recurrence 8 months later, which metastasized approximately 1 year postrecurrence. It is not possible to draw a firm conclusion with only one paired sample; however, these observational data are consistent with the overall cohort analysis, whereby miR-211 was the sole miRNA to identify metastatic UM.

## Discussion

A recent review of clinical studies validated the high degree of diagnostic accuracy of a multistep clinical diagnostic guideline for choroidal nevi.^18^ The suggested guideline lists risk factors for transformation as lesion thickness (>2 mm), subretinal fluid, symptoms (e.g., decreased vision), orange lipofuscin, tumor margin within 3 mm of the optic disc, ultrasonographic hollowness, and lack of halo. The presence of three risk factors confers a 50% chance of malignant transformation. Notably, a lesion presenting with more than 2-mm thickness, symptoms, and location near the disc has a 69% risk of growth.^3^ Once diagnosed with a choroidal nevus with “high-risk” features, or following treatment of noninvasive UM, patients and treating clinicians alike are left with some uncertainty over the malignant propensity of these lesions—as such there is a great need for a biomarker to confirm initial diagnosis as well as to detect early signs of malignant change and metastatic progression.

Herein, we describe a panel of six circulating miRNAs that may fulfill this clinical need. This panel offers a high degree of precision to the diagnosis of UM, achieving 93% sensitivity and 100% specificity. The clinical utility of this panel is evident at the nevus stage of diagnosis, with circulating miRNA expression levels accurately distinguishing benign lesions from UM (Fig. and Table 2). Following subsequent validation studies in larger longitudinal study cohorts, this panel could be used as a companion diagnostic tool to assist in clinical decision making. Such as, at initial presentation, to inform a decision between close observation and monitoring a suspicious borderline lesion, or treatment to achieve early local tumor control. Furthermore, this panel may be well-suited for a minimally invasive tool to serially monitor patients initially presenting with benign lesions to identify signs of progression at subsequent follow-up visits.

Since commencement of this study, there have been a small number of studies investigating the utility of circulating miRNAs, as well as a soluble oncoprotein (c-Met), for UM detection.^12^–^14^,^19^ A common miRNA to all studies is miR-146a, which was found to be upregulated in UM patient serum/plasma as well as archival tissues.^12^–^14^ In our study, we too confirmed that miR-146a was increased in the serum of localized (*P*[cor] = 0.0003) and metastatic (*P*[cor] = 0.0019) UM patients as compared with individuals with uveal nevi. In these prior studies, miR-146a was proposed to be circulating marker of UM, and our data support the use of miR-146a as a member of a panel to increase diagnostic accuracy.

A limitation of this study is the small sample size of participants with metastatic UM or choroidal nevi, a larger cohort, which would enable thorough investigation of the clinical risk features that have been identified.^3^ In addition, without serial blood draws (except for one individual), this panel was unable to predict or detect recurrence. Low miR-204 expression did however have a significant association with poor prognosis (Supplementary Fig. S5).

### Conclusion

In both cutaneous and uveal melanoma, early detection of primary lesions offers the best hope to prevent progression to metastatic disease. This is a difficult task in the case of UM as uveal nevi may remain stable for the duration of a patient's life but require regular monitoring by a specialized ocular oncologist. Currently, there are no biomarkers available for use in clinical practice that offer a degree of diagnostic certainty. The six-miRNA panel described herein offers promise in identifying early signs of malignant transformation and progression detection. However, further investigation and validation in larger prospective cohorts is warranted.

## Supplementary Material

Supplement 1
