# Supplementary material for: A Panel of Circulating MicroRNAs Detects Uveal Melanoma With High Precision
Source: Transl Vis Sci Technol. 2019 Nov 14;8(6):12. doi: 10.1167/tvst.8.6.12 (PMC6855372; doi:10.1167/tvst.8.6.12)

**Supplementary Material**

**A panel of circulating microRNAs detects uveal melanoma with high precision**

Mitchell S. Stark, PhD ^1*^; Elin S. Gray, PhD ^2,3^; Timothy Isaacs, MBBS, FRCOphth, FRANZCO ^3,4,5,^; Fred K. Chen, MBBS, PhD, FRANZCO ^3,4,6^; Michael Millward, MD ^7,8^; Ashleigh McEvoy, PhD ^2^; Pauline Zaenker, MSc ^2^; Melanie Ziman, PhD ^2,9^; H. Peter Soyer, MD ^1,10^; William J. Glasson, MBBS, FRANZCO, FRACS, FRCOphth ^11^; Sunil K. Warrier, MBBS, FRANZCO ^11^; Andrew L. Stark, BSc (Hons) ^11^; Olivia J. Rolfe, BSc ^11^; Jane M. Palmer, RN ^12^_;_ Nicholas K. Hayward, PhD ^12^

^1^ Dermatology Research Centre, The University of Queensland Diamantina Institute, The University of Queensland, Woolloongabba, QLD 4102, Australia.

^2^ School of Medical and Health Sciences, Edith Cowan University, Joondalup, Western Australia, Australia

^3^ Centre for Ophthalmology and Visual Science, The University of Western Australia, Crawley, Western Australia, Australia

^4^ Department of Ophthalmology, Royal Perth Hospital, Perth, Western Australia, Australia

^5^ Perth Retina, West Leederville, Western Australia, Australia

^6^ Lions Eye Institute, Nedlands, Western Australia, Australia

^7^ School of Medicine and Pharmacology, The University of Western Australia, Crawley, Western Australia, Australia

^8^ Department of Medical Oncology, Sir Charles Gairdner Hospital, Nedlands, Western Australia, Australia

^9^ School of Biomedical Science, The University of Western Australia, Crawley, Western Australia, Australia.

^10^ Department of Dermatology, Princess Alexandra Hospital, Brisbane, Queensland, Australia

^11^ Queensland Ocular Oncology Service, The Terrace Eye Centre, Brisbane, Queensland, Australia

^12^ QIMR Berghofer Medical Research Institute, Brisbane, Queensland, Australia

***** Corresponding author

Dr Mitchell S. Stark

Dermatology Research Centre,

The University of Queensland Diamantina Institute,

Level 5, Translational Research Institute,

37 Kent Street, Woolloongabba, Brisbane,

QLD 4102, Australia

Phone: +61 7 3443 8027

Fax: +61 7 3443 7779

Email: [m.stark@uq.edu.au](mailto:m.stark@uq.edu.au)

**Supplementary Figures**

**Supplementary Figure 1**


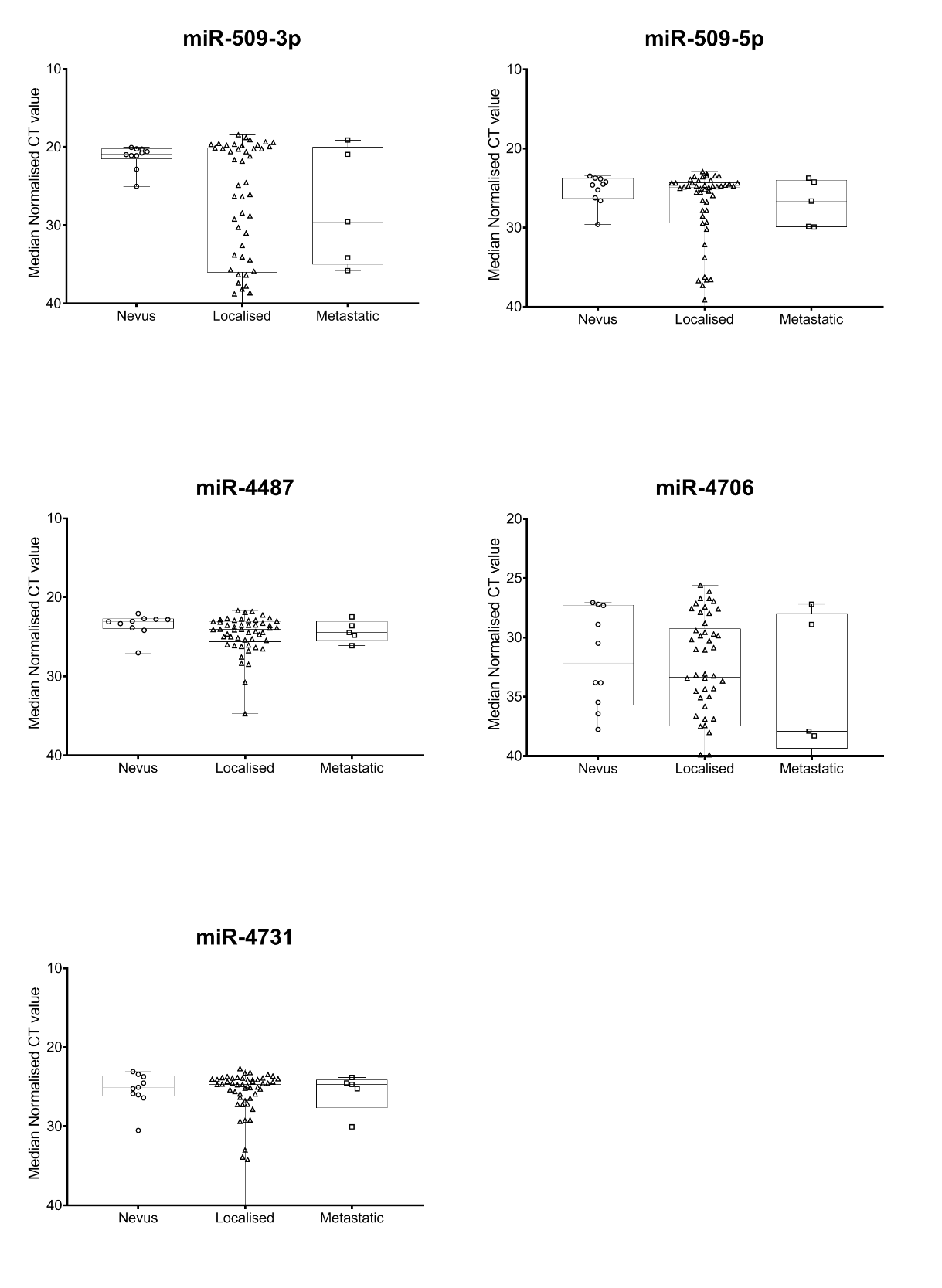
Box and Whisker plots (Min to Max) represent all data points not represented in Table 2 for the miRNAs not did not reach significance (ANOVA *p*>0.05) across the cohorts.

**Supplementary Figure 2**


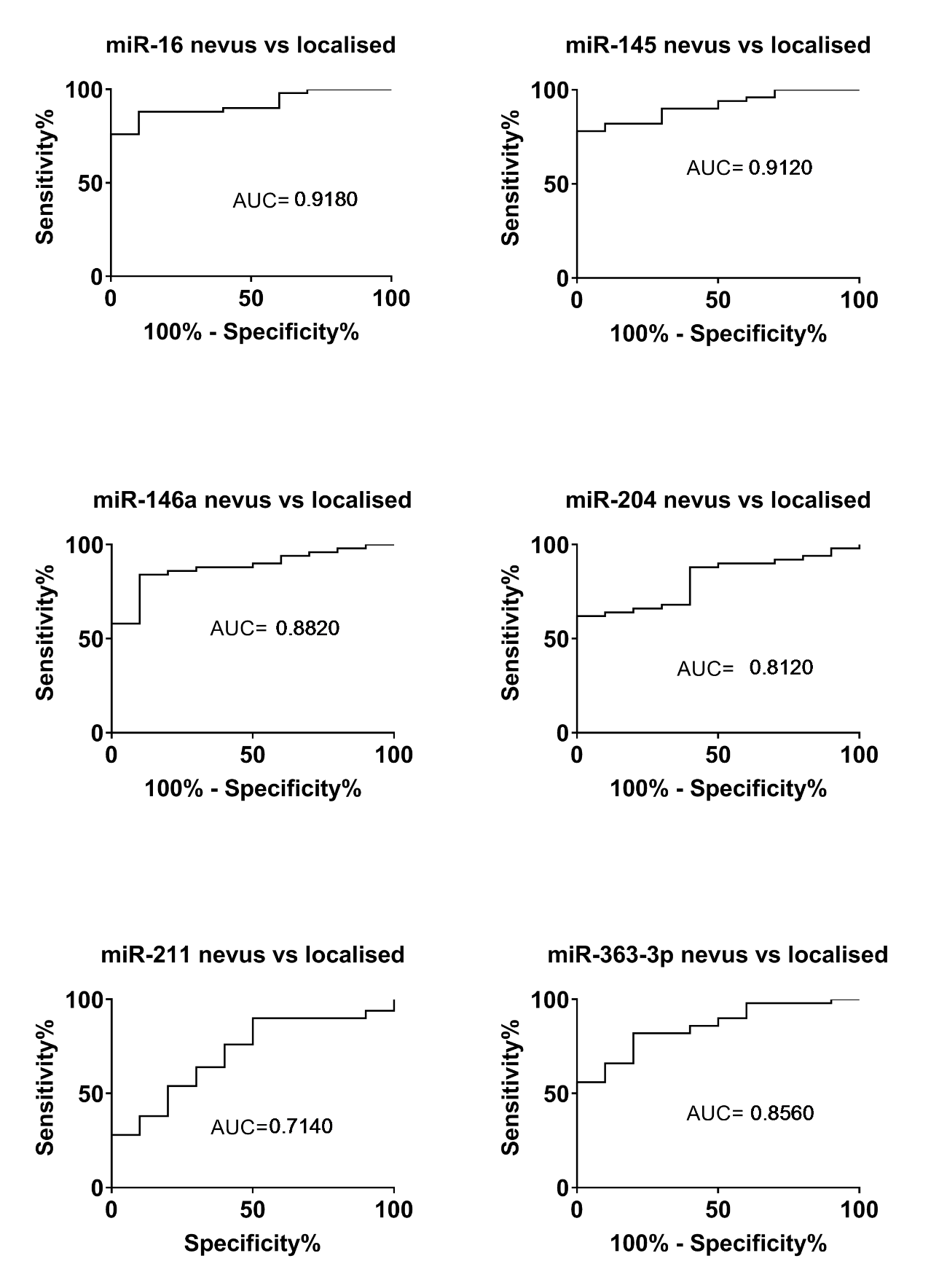
ROC curves derived from the data presented in Table 2 for the six-miRNA panel that were significantly different (*p*<0.05) when uveal nevi *cf.* patients with localised uveal.

**Supplementary Figure 3**

ROC curves derived from the data presented in Table 2 for the six-miRNA panel that were significantly different (*p*<0.05) when uveal nevi *cf.* patients with metastatic. miR-204 was known to be non-significant (*p*>0.05; Table 2) and shows an uninformative AUC score (0.52).


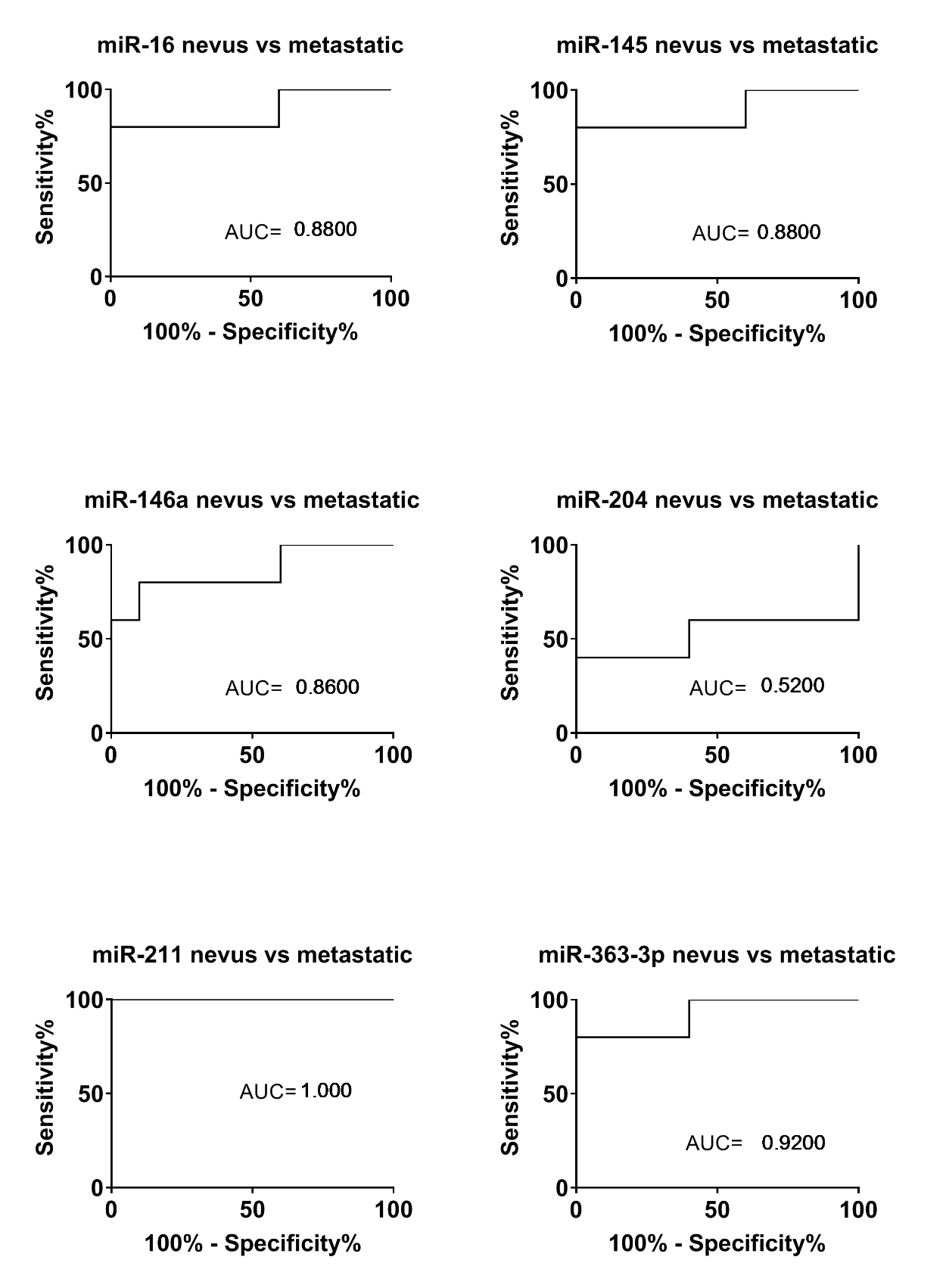


**Supplementary Figure 4**

ROC curves for miR-211 highlighting its diagnostic utility in identifying metastatic disease.


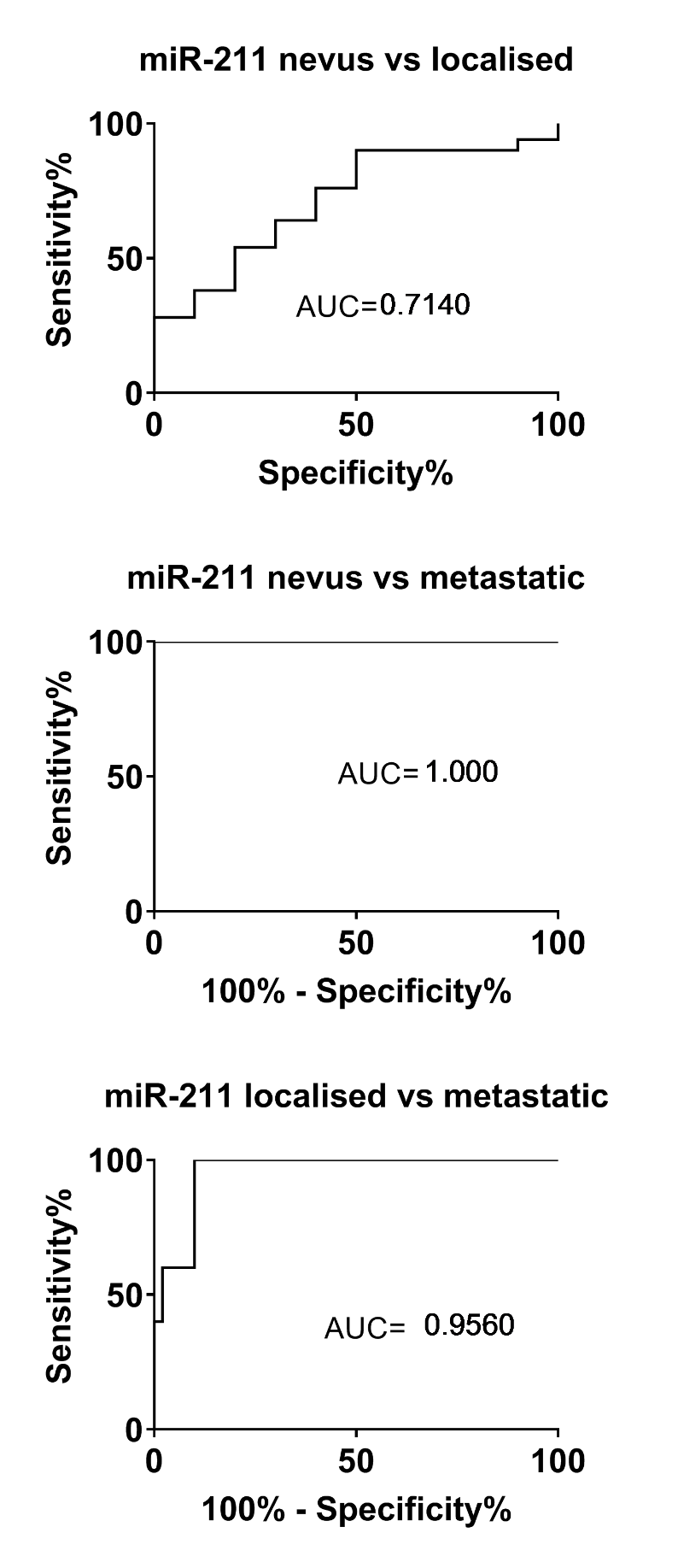


**Supplementary Figure 5**

Kaplan Meier survival analysis (overall survival) for circulating miR-204 and miR-211 expression levels.


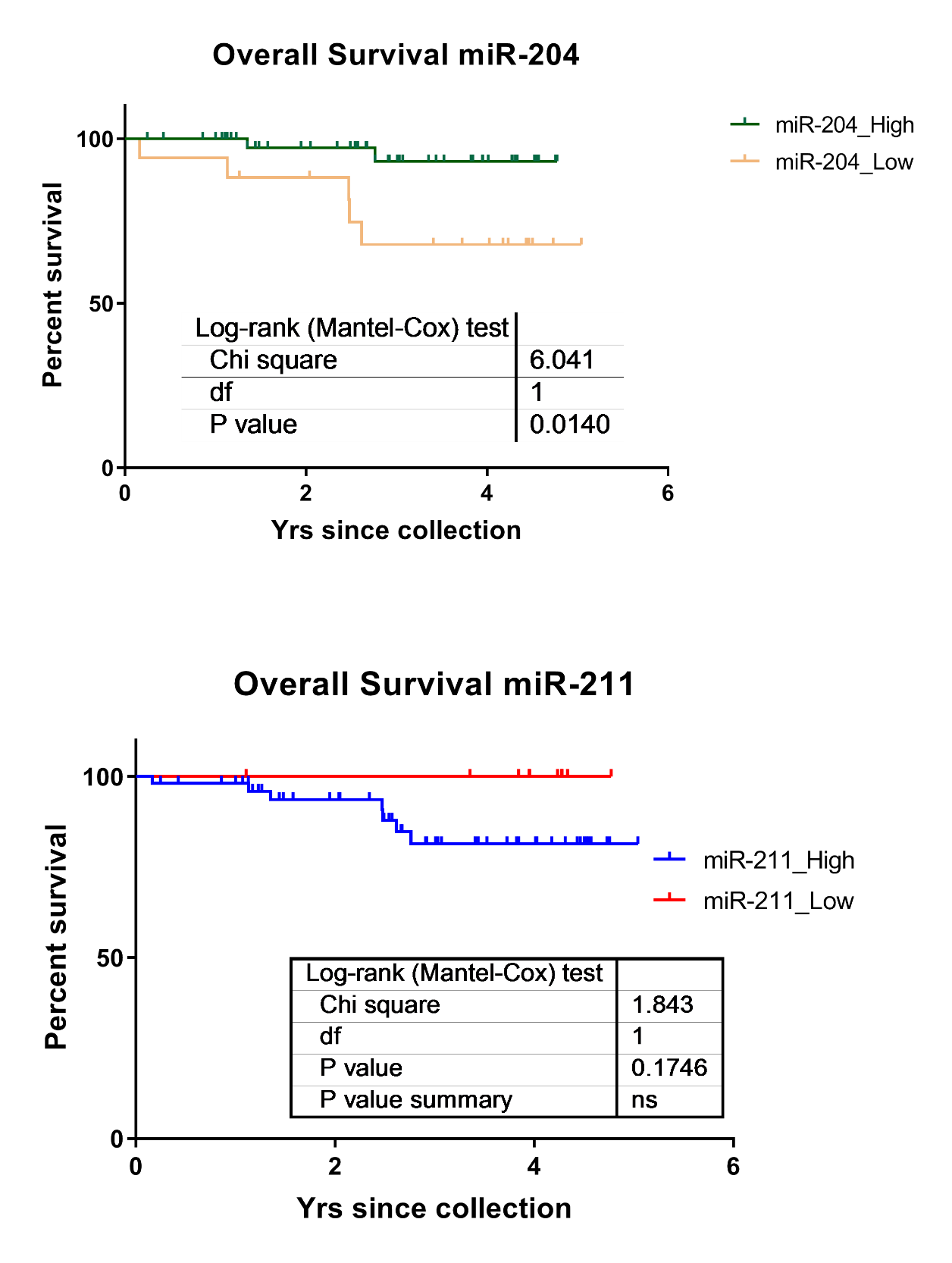

Supplement: Supplement 1 [file tvst-08-05-22_s01.docx]
